# A comparative analysis of biogas production from tomato bio-waste in mesophilic batch and continuous anaerobic digestion systems

**DOI:** 10.1371/journal.pone.0248654

**Published:** 2021-03-17

**Authors:** Árpád Szilágyi, Attila Bodor, Norbert Tolvai, Kornél L. Kovács, László Bodai, Roland Wirth, Zoltán Bagi, Ágnes Szepesi, Viktória Markó, Balázs Kakuk, Naila Bounedjoum, Gábor Rákhely

**Affiliations:** 1 Department of Biotechnology, University of Szeged, Szeged, Hungary; 2 Institute of Environmental Sciences, University of Szeged, Szeged, Hungary; 3 Institute of Biophysics, Biological Research Centre, Szeged, Hungary; 4 Department of Oral Biology and Experimental Dental Research, University of Szeged, Szeged, Hungary; 5 Department of Biochemistry and Molecular Biology, University of Szeged, Szeged, Hungary; 6 Department of Plant Biology, University of Szeged, Szeged, Hungary; Tsinghua University, CHINA

## Abstract

Annually, agricultural activity produces an enormous amount of plant biomass by-product. Many studies have reported the biomethane potential of agro-industrial wastes, but only a few studies have investigated applying the substrates in both batch and continuous mode. Tomato is one of the most popular vegetables globally; its processing releases a substantial amount of by-product, such as stems and leaves. This study examined the BMP of tomato plant (*Solanum lycopersicum* Mill. L. cv. Alfred) waste. A comparative test revealed that the BMPs of corn stover, tomato waste,and their combination were approximately the same, around 280 mL methane/g Volatile Solid. In contrast, the relative biogas production decreased in the presence of tomato waste in a continuous mesophilic anaerobic digestion system; the daily biogas productions were 860 ± 80, 290 ± 50, and 570 ± 70 mL biogas/gVolatile Solid/day in the case of corn stover, tomato waste, and their mixture, respectively. The methane content of biogas was around 46–48%. The fermentation parameters of the continuous AD experiments were optimal in all cases; thus, TW might have an inhibitory effect on the microbial community. Tomato plant materials contain e.g. flavonoids, glycoalkaloids (such as tomatine and tomatidine), etc. known as antimicrobial and antifungal agents. The negative effect of tomatine on the biogas yield was confirmed in batch fermentation experiments. Metagenomic analysis revealed that the tomato plant waste caused significant rearrangements in the microbial communities in the continuously operated reactors. The results demonstrated that tomato waste could be a good mono-substrate in batch fermentations or a co-substrate with corn stover in a proper ratio in continuous anaerobic fermentations for biogas production. These results also point to the importance of running long-term continuous fermentations to test the suitability of a novel biomass substrate for industrial biogas production.

## Introduction

There is a growing interest in renewable energy sources due to the depletion of fossil fuels and their negative effects on the environment. One of the most common non-conventional energy carriers is biogas produced in anaerobic digestion (AD) processes. A wide variety of biomasses, such as maize or grass silage, crop plant and agricultural by-product, wastewater sludge, food processing by-products, domestic organic waste, and manure, have been used as substrates in biogas plants [1–3]. The stability and productivity of the biogas plants could be affected by diverse substrates. Moreover, the microbial community composition fluctuates and varies with the substrates [1].

AD has an increasing role in the agricultural sector for the energetic utilization of organic wastes [4]. Approximately 5 billion hectares are used for agricultural production worldwide [5], generating large volumes of plant biomass by-products, such as orange, onion, and potato peels, green plant residues, and tomato pomace [6].

Many studies have reported that various kinds of agricultural by-product, e.g., cabbage, capsicum, pepper, cucumber, eggplant, and tomato residue, are suitable for biogas production [4, 7, 8].

A lignocellulosic biomass’ energy potential depends on its composition, such as the lignin, hemicellulose, and cellulose ratio, and the microbial community formed or designed to convert these fibers to energy carrier. However, various plant species might produce antimicrobial agents that negatively affect the microbial activities during e.g. AD [9–11]. Moreover, the methane yield can be influenced by parameters, such as temperature, pH, volatile fatty acids (VFAs), ammonia, macro- and micronutrients, and potentially toxic compounds. The toxic or inhibitory compounds could modify microorganisms’ activity [2, 12].

Therefore, it will be useful to investigate the optimal mix of substrates and the conditions of fermentation. While the biomethane potential (BMP) of many wastes in batch fermentation experiments have been tested, there are just a few studies comparing the batch fermentation to continuous fermentation [4, 7]. Batch fermentations are suitable in estimating a substrate’s methane yield and biodegradability, but they do not provide information about the long-term effect of the substrate on the fermentation.

Several standard methods, such as DIN 38414 TL8, ASTM D 5210, ISO 11734, ISO 14853, ISO 15985, and VDI 4630, are used to evaluate BMP [13]. These standards prescribe the conditions of fermentation, including the amount of inoculum, substrates, and medium, the definition of the necessary controls, blanks, and replicates, and the specification of the experimental setup [13]. In continuous AD system, the daily feeding of reactors, the removal of fermentation liquid, the harvest of biogas, and the monitoring of the fermentation parameters are carried out periodically over the long term. In this experimental setup, the durable effect of the applied substrate could be examined [14].

Also, the rapid expansion of “omics” approaches has enabled a better understanding of the AD process. Analyzing the structure, composition, and activity of the involved microbial communities and combining the metagenomics and culture-dependent methods are necessary to gain a deeper insight into the fermentation processes, identify the key factors involved in the optimization of the AD process parameters and enhancement of the biogas yield from a more diversified group of plant biomass waste substrates [15, 16].

Tomato (*Solanum lycopersicum*) is one of the most widely cultivated vegetable crops globally [7]. During its traditional or greenhouse cultivation, harvesting, and industrial processing, most of a tomato plant remains unused, producing a large quantity of waste consisting of peels, seeds, stems, and leaves that reached 473,989 tons in 2017 [17]. Greenhouse cultivation alone produces 15 tons/ha/year of tomato plant waste [18]. Tomato stems and leaves contain various bioactive substances, such as phenols, flavonoids, and glycoalkaloids, such as tomatine and tomatidine, which have possible antimicrobial, antiviral, and antifungal effects [9]. Traditionally, these by-products are returned to the global cycles of goods by composting or burning [19, 20] or discarded to landfill.

Managing these by-products has become a global environmental and economic issue. However, tomato waste is also one of the most underutilized sources of renewable energy [21]. Nowadays, most studies focus on utilizing tomato crops, seeds, and peels for biogas production or carotenoid extraction. Many studies investigated the utilization of various kinds of tomato waste, such as those from groceries, processing plants, tomato sauce, and tomato puree [22–26]. There are only few reports on the bioenergetic utilization of stems and leaves from tomato wastes [7, 26–29]. A previous study evaluated the BMP potential of two different tomato plant residue, mostly stems and leaves; however, the stems and leaves’ effects on continuous fermentation were not investigated [8].

This study compares the BMPs of tomato stems and leaves–potentially useful substrates–in batch and continuous AD. We applied tomato waste as mono- and co-substrate in both types of fermentation and followed the process parameters with standard analytical techniques. The metagenomic approach was used to monitor the variations in the microbial communities caused by the substrates. This study is the first attempt to carry out a complex investigation of tomato waste (stems and leaves), as a mono- or co-substrate, in batch and continuous AD systems.

## Materials and methods

### Substrate specification

Tomato (*S*. *lycopersicum* Mill. L. cv. Alfred) plant waste (TW) was obtained from the Department of Plant Biology at University of Szeged. Dry corn stover (CS), provided by the Faculty of Agriculture, University of Szeged, was used as the positive control in both batch and continuous AD experiments.

Both, tomato stems and leaves and corn stover came from field experiments and were recovered after the harvest. The air-dried plant materials were milled and sieved with an electric grinder (Retsch SM 100, Haan, Germany). The maximum particle size was 2 mm. In the co-fermentation experiments, CS and TW were combined at a 7:3 ratio (CoS) based on volatile solid and C/N measurements.

### Total solid, volatile solid, and C/N ratio measurements

To determine the dry matter content (total solid, TS), the plant materials were kept at 105°C until their weight became constant. The volatile solid (VS) content was determined by placing the dried residues in an incinerator at 550°C for 2 hours. An Elementar Vario MAX CN (Elementar Group, Hanau, Germany) analyzer was used to determine the C/N ratio of the substrates. The temperature of the combustion and post combustion tube was set to 900°C, the temperature of the reduction tube was set to 830°C. After the samples were burnt in the combustion tube the water vapour was separated by a specific adsorption column containing Sicapent (Merck KGaA, Darmstadt, Germany). The components were detected with a thermal conductivity detector. Helium (5.0) was the carrier and flushing gas (Messer Group, Bad Soden, Germany).

### Fiber analysis

FIWE 3 Fiber Analyzer (VELP Scientifica, Usmate, Italy) was used to determine the fiber composition of the applied substrates [30].

### Inoculum

The AD inoculum was collected freshly from a biogas plant (Zöldforrás Ltd., Szeged, Hungary), which operated with a mixture of pig slurry and maize silage under mesophilic conditions. Prior to utilization, the sludge was filtered through a 2 mm filter to remove particles larger than 2 mm and undigested plant materials.

### Batch fermentation

The biomethane potential (BMP) tests of the CS (positive control), TW, and CoS were performed in batch fermentation in 125 mL Wheaton glass serum bottles (Merck KGaA, Darmstadt, Germany). Based on the VS contents, the substrate to the inoculum ratio was set to 2:1 (VDI I.) and 1:1 (VDI II.). Blank samples containing only water and inoculum were prepared to measure the background methane formation. The bottles were sealed with rubber septa and the airspaces were flushed with nitrogen gas (Purity: 5.0, Messer group, Bad Soden, Germany) for 10 minutes. These experiments were performed in triplicates following the VDI 4630 standard. The fermentations were carried out under mesophilic condition (37.0°C ± 0.5°C) in the final volume of 40 mL. The reactor’s initial pH was 7.5 ± 0.15. Methane content in the headspace of the bottles was measured daily for 30 days. 100μl samples were taken with a Gastight Hamilton syringe and analyzed by gas chromatography (GC) (Section Gas chromatography analysis). Each sample was stirred manually before gas analysis. All fermentations were performed in triplicates.

### Continuous fermentation

Both tomato waste as a mono-substrate (TW) and co-substrate (CoS) were also tested in continuous fermentation. The fermentations were carried out in 5 L, continuously stirred tank reactors (CSTRs) [31]. The reactors were filled with the inoculum and then operated under a mesophilic (37°C) condition until the residual biogas production dropped to zero. We subsequently started to feed the reactors daily (OLR: 1 g VS/L; particle size: <2mm). The volume and methane content of the biogas were measured every day; the various fermentation parameters, such as pH, electroconductivity, Volatile Organic Acid/Total Inorganic Carbon (VOA/TIC), ammonium-ion cc., were recorded weekly. The volume of the produced biogas was determined by Brooks gas flow meters (5860S/BA1KA0BA0BA1B1, Brooks Instrument, Hatfield, USA) directly connected with the reactors. The methane content of the generated biogas was measured (Section Gas chromatography analysis).

### Gas chromatography analysis

The CH_4_ content was measured daily by an Agilent 6890N gas chromatograph (Agilent Technologies, Santa Clara, United States). The GC was equipped with a HP Molesive 5 Å column (length 30 m, I.D. 0.53 mega bore, film 25 μm) and a thermal conductivity detector. The flow-rate of Argon 5.0 (Linde Group Hungary, Budapest, Hungary) carrier gas was set to 16.8 mL/min. Split injection mode was applied at 0.2:1 and 150°C. During batch fermentation, the headspaces of the bottles were flushed with nitrogen gas (Messer Group, Bad Soden, Germany) for 10 minutes after each sampling and measurement in the case of batch fermentation. In continuous AD systems, gas samples were taken from the reactor’s headspace in every day into a 15 mL gas tight serum bottle and the methane measurements were carried out as we described above. The methane calibration was performed with certified gas mixture ((5% CO_2_; 5% CH_4_; 5% H_2_; 85% N_2_) Linde plc., Dublin, Ireland)). For the sampling and injection, Hamilton sample lock syringe was used (Merck KGaA, Darmstadt, Germany).

### Fermentation parameters

The pH of the samples was measured with a Radelkis OP-211/2 digital pH meter (RADELKIS Kft, Budapest, Hungary). The volatile organic acid and total acid capacity (VOA/TIC) of the samples were determined using a Pronova FOS/TAC 2000 instrument.

### High throughput DNA sequencing

A 150 mg sample from each reactor was collected for total community gDNA purification at the start of fermentation (inoculum) and on the 40^th^ day to determine the digester’s microbial compositions. The samples were stored at −20°C for later use. The extractions were performed with a Quick-DNA Fecal/Soil Microbe Kit (Zymo Research Corporation, Irvine, USA). The DNA was first quantified with a Qubit 4.0 fluorimeter (Invitrogen, Waltham, USA), and then, its integrity was examined on 1% agarose gel.

The 16S Metagenomic Sequencing Libraries were prepared according to the Illumina’s protocol. The V3-V4 region of 16S rDNA was amplified by PCR (Illumina, San Diego, USA), with the Illumina_16S_341F and Illumina_16S_805R primer pair [32]. A 25-μL PCR reaction mixture contained 12.5 ng of genomic DNA, 2x KAPA HiFi HotStart Ready Mix, and 0.2 μM of each primer. The PCR’s parameters include initialization at 95°C for 3 min, and 25 cycles of denaturation, annealing, and extension at 95°C, 65°C and 72°C (each for 30 sec), and final elongation at 72°C for 5 minutes. The PCR products were purified, analyzed, indexed, then the libraries were validated and sequenced (Illumina MiSeq platform, MiSeq® Reagent Kit v3 (600 cycles), by Seqomics Ltd., Mórahalom, Hungary). The sequencing data is available on the NCBI Sequence Read Archive (Submission number: SAMN13231577).

### Bioinformatics methods for metagenomic analysis

The trimming and quality filtering of the sequencing reads was carried out using the *DADA2’s* [33] *filterAndTrim* function (parameters: truncLen = c(240,220), maxN = 0, maxEE = c(2,2), truncQ = 2, rm.phix = T, ctrimLeft = c(50, 55)) in the R environment (R version 3.5.3, *Great Truth*) [34]. The trimmed and quality-filtered reads were uploaded to the One Codex webserver and classified by the Targeted Loci Database [35]. The phylum and genus-level results were downloaded in CSV format and imported into the R package. The Principal Component Analysis was carried out with *FactoMineR* [36] and visualized with *factoextra* [37]. The *phyloseq* package was used for richness estimation and visualization. The statistical tests were carried out with the DESeq2 (test = "Wald", fitType = "parametric") [38, 39]. The differences were considered significant by adjusting a p-value threshold to 0.01. The results were visualized with ggpubr [40].

### Tomatine and tomatidine inhibition test in batch mode

The effects of tomatine and tomatidine were tested in batch experiments using α-cellulose as a substrate. The fermentations were conducted according to the VDI 4630 standards (Sections Inoculum, Batch fermentation and Continuous fermentation; https://www.vdi.de/en/home). 0.1, 1, 10, 100 μg tomatine or tomatidine were dissolved in DMSO and put into the reactors. Positive and solvent controls were used as well. Positive control contained inoculum, water and α-cellulose; the solvent control contained inoculum, water, α-cellulose and DMSO without tomatine or tomatidine. The methane production was measured daily for 16 days, till the methane formation dropped to zero.

### Phytotoxicity test

Lettuce is a good model plant for monitoring phytotoxic compounds [41]. The germination/elongation tests of lettuce seeds (*Lactuca sativa L*.) were performed to estimate the fermentation effluents’ phytotoxicity. Germination inhibition and root elongation inhibition were conducted on a thin layer of filter paper placed on a Petri dish (90 *× 20 mm). Each plate was supplemented with 1, 3, or 5 v/v% fermentation sludge solution in distilled water; an identical volume of distilled water acted as a control. 30 seeds were placed on each plate. The Petri dishes were incubated at 25°C with lids in the dark. The appearance of radicles indicated germination. Measurements were performed after 0, 1, 2 and 3 days of sludge treatments. The number of germinated seeds and the length of the radicles were determined by ImageJ software [42].

### Statistical analysis

The data were analyzed by GraphPad Prism 8 Software, and Dunnett’s multiple comparison test was applied in every case.

## Results and discussion

### Batch fermentation of corn stover, tomato waste, and co-substrate

The BMP of tomato stem and leave residues were determined in batch fermentation experiments. The tomato wastes were used as mono-substrate (TW) and co-substrate (CoS) with CS. Based on CS and TW’s VS-, carbon- and nitrogen contents, CS and TW were mixed into CoS at a 0.7:0.3 ratio. Before fermentation, the VS, TS, carbon content, nitrogen content, C/N ratio, and fiber composition of the substrates was determined (Tables 1 and 2).

**Table 1:** Different parameters of the used substrates.

	TS (%)	VS (%)	C content (%)	N content (%)	C/N ratio	particle size [mm]
Corn stover (CS)	92.80 ± 0.25	91.10 ± 0.32	44.02 ± 1.31	1.1 ± 0.03	39.88 ±0.08	<2
Tomato waste (TW)	92.12 ± .012	83.74 ± 0.05	37.27 ± 0.34	1.77 ± 0.05	21.09 + 0.77	<2
Co-substrate (CoS)	92.18 ± 0.13	88.05 ± 0.40	41.03 ± 0.94	1.46 ± 0.21	28.46 ± 3.29	<2

TS-Total Solid, VS- Volatile Solid, C content-carbon content, N content- nitrogen content, C/N ratio-carbon/nitrogen ratio

**Table 2:** Fiber composition of the used substrates (%).

	Solubles	Hemicellulose	Cellulose	Lignin
TW	56.57 ± 1.2	9.9 ± 0.13	26.86 ± 1.82	6.69 ± 0.49
CS	28.2 ± 2.34	23.00 ± 1.44	32.12± 1.93	14.23 ± 1.71
CoS	36.57 ± 1.5	19.06 ± 0.71	30.46± 0.4	11.81 ± 0.99

TW-Tomato waste, CS-Corn stover, CoS-Co-substrate

The TS contents of TW, CS, and CoS are very similar, while the VS content of TW is 8% lower than that of CS; however, the CS has a C/N ratio at least two times higher than those of TWs (Table 1). The optimal C/N ratio for an AD process ranges from 20 to 30 [43, 44]. The C/N ratios of CS, TW and CoS fall into the optimal C/N range. The main fiber components of the CS, TW, and CoS, i.e., solubles, hemicellulose, cellulose, and lignin, were also determined (Table 2); CS had a higher content of hemicellulose, cellulose, and lignin than TW.

On the other hand, TW’s soluble content was approximately two times higher than that of CS. The lignin content is important to a substrate’s digestibility. A cross-linked, heterogeneous biopolymer, lignin is hard to degrade; it prevents microorganisms and their enzymes from accessing the cellulose and hemicellulose [45]. Therefore, TW’s lower lignin and higher soluble content make it more accessible to microbial utilization.

**Fig 1:**
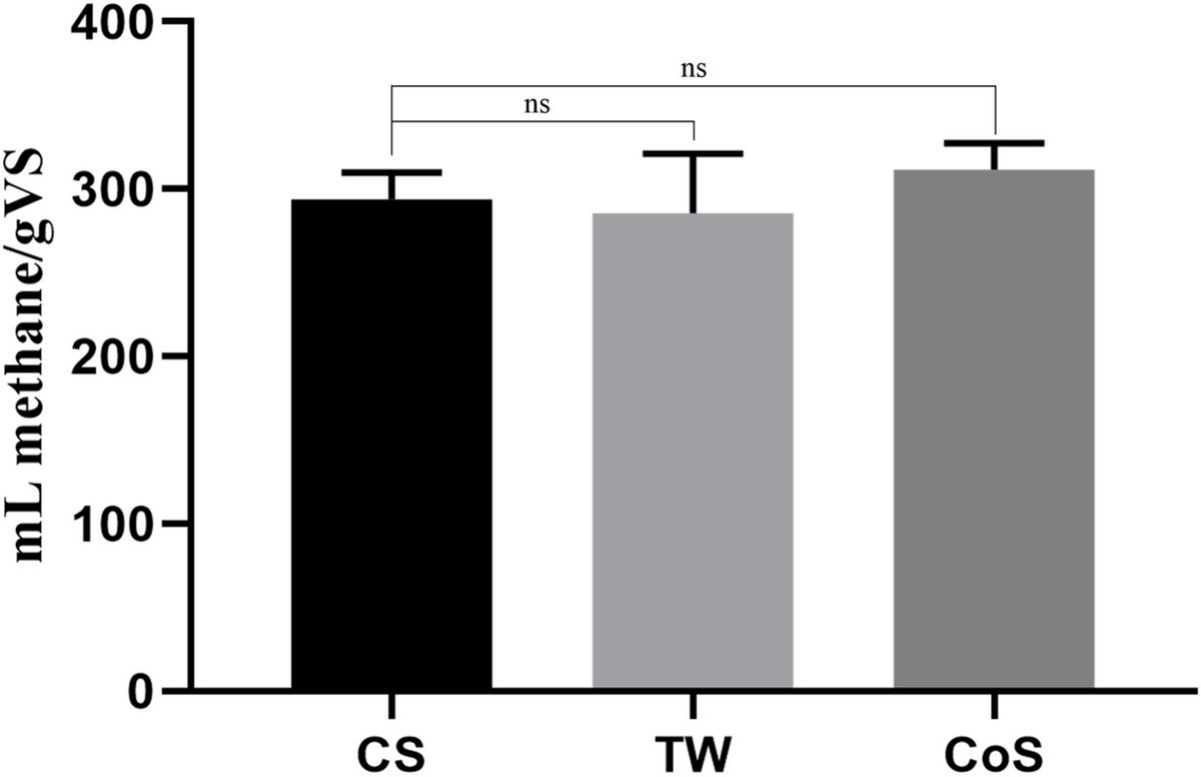
The cumulative biogas yield of the batch experiments. The substrates used were corn stover (CS), tomato waste (TW), and co-substrate (CoS, a mixture of CS and TW) with simple (VDI I.) and double (VDI II.) substrate loads. There are no significant differences between the cumulative biogas yield of substrates according to the Dunnett’s multiple comparison test (n = 3, p ≤ 0.05).

The cumulative methane yields from CS, TW, and CoS (both VDI I. and VDI II. systems) were statistically indistinguishable, they varied between 245±18 and 289±15 mL/gVS (Fig 1). Tomato crop residue (stems, leaves, vines and residual fruit) was previously tested in batch experiments by Li et al. where the biogas yield was 214.8 mL/gVS with 57.9% methane content (124.4 mL methane/gVS [46], which was substantially lower than our yields.

The experiments suggest that both TW and CoS are promising substrates for AD.

We also noted that TW’s methane production rate was higher than CS in the first 5 days, which might be attributed to TW’s higher soluble content and lower lignin concentration (S1 Fig).

### Anaerobic digestions of TW, CS, and CoS in a continuous system

TW’s persistent effect on fermentation was tested in continuous AD experiments using mono- (TW) and co-substrate (CoS). In these experiments, CS was also used as a positive control. First, the CSTR were fed with CS, TW, and CoS for three weeks to adapt their microbiota to the substrates. After the adaptation phase, the biogas production and methane contents were measured daily for 56 days.

**Fig 2:**
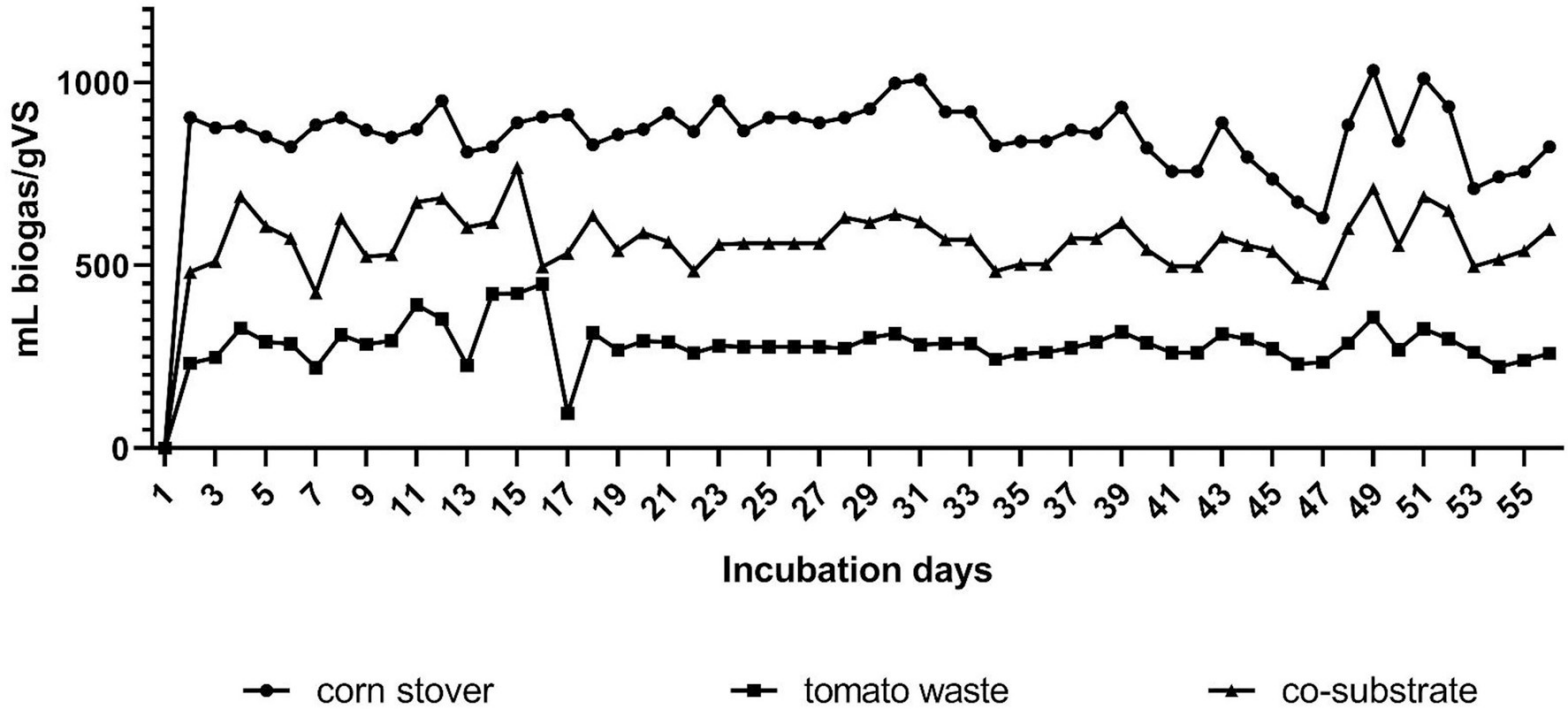
The daily biogas production of continuous experiments. The substrates used were corn stover (CS), tomato waste (TW), and co-substrate (CoS, a mixture of CS and TW). According to the statistical analysis, there was a significant difference between the CS and TW, as well as CS and CoS. n = 56, p ≤ 0.0001 (Dunnett’s multiple comparison test).

The daily biogas yields of the continuous AD were 860 ± 80, 290 ± 50, and 570 ± 70mL/(gVS * d) with CS (CSTR_CS_), TW (CSTR_TW_), and CoS (CSTR_CoS_), respectively (Fig 2); the methane concentrations were comparable at 46.01 ± 3.56 (CSTR_CS_), 46.91 ± 3.77 (CSTR_TW_), and 48.10 ± 3.96% (CSTR_CoS_). The volume of the daily produced biogas drastically dropped in the presence of TW, by 66.28%, in the case of TW/CS and by 33.72% in the case of CoS/CS. Throughout the CSTR experiments, the fermentation parameters, i.e., pH, VOA/TIC, conductivity and NH_4_^+^ concentrations, remained within the optimal ranges. The pH varied between 7.20 and 7.92. The VOA/TIC value was between 0.1–0.3, and the NH_4_^+^ concentrations were less than 1.2 g/L.

The CSTR results diverge from our batch fermentation experiments and a previous study on BMP [8]. Since both approaches had the other fermentation parameters in the optimal range, their difference might be due to the presence of TW component that reduced biogas production in the CSTR system. A similar phenomenon was also observed by Li and colleagues in solid state, hemi-solid state and liquid AD systems using tomato residues (stalks, leaves and other wastes) [47]. According to their conclusions, the higher loading of tomato residues might cause stress leading to lower methane production. However, TW, including stems and leaves, contains antimicrobial compounds, such as flavonoids, phenols, vitamins, tomatine, and tomatidine [9–11]. The continuous input of these substances might influence the composition of the AD microbiota. Therefore, we carried out a 16S metagenomic analysis of CSTRs (Section Microbial composition in the continuous fermenters) and tested the effect of tomatine and tomatidine in batch fermentation experiments (Section Tomatine and tomatidine toxicity).

### Microbial composition in the continuous fermenters

#### Bacterial community

The microbial communities in continuous fermentation were analyzed on day 0 (inoculum) and 40 of the experiment by 16S amplicon metagenomic analyses. The statistics of the crude and processed sequencing reads are shown in Table 3.

**Table 3:** Statistic of the used reads.

readcounts						
Samples	input	filtered	denoisedF	denoisedR	merged	nonchim
CS/1	56791	47856	46381	46654	18289	17022
CS/2	52597	44319	40952	41460	18218	16084
CS/3	52019	42515	39183	39811	17191	15185
TW/1	54535	46605	45735	45980	21142	20136
TW/2	56333	48137	47184	47455	22385	21365
TW/3	50528	42531	41733	41961	19706	18785
CoS/1	57528	48167	46768	47023	26622	23788
CoS/2	63492	51770	50298	50674	29101	26267
CoS/3	60378	51086	49718	50006	28762	26017

CS, TW and CoS-fermentors fed with corn stover, tomato waste and co-substrate, respectively. 1,2,3 are the technical paralell samples

The Shannon and Simpson indices were calculated to characterize and compare the microbial diversities (alpha diversity) in the various fermentations (S2 Fig). CSTR_TW_’s Shannon and Simpson indices were much lower than those of CSTR_CS_ and CSTR_CoS_, suggesting that the microbial communities in CSTR_CS_ or CSTR_CoS_ fermentations exhibited substantially higher diversities than CSTR_TW_. Also, CSTR_CoS_’ diversity indices were more elevated than CSTR_TW_’s, indicating an increased in diversity when CSTRs were fed with both substrates. In addition, these results are consistent with the finding that TW contains antimicrobial compounds [9, 11].

**Fig 3:**
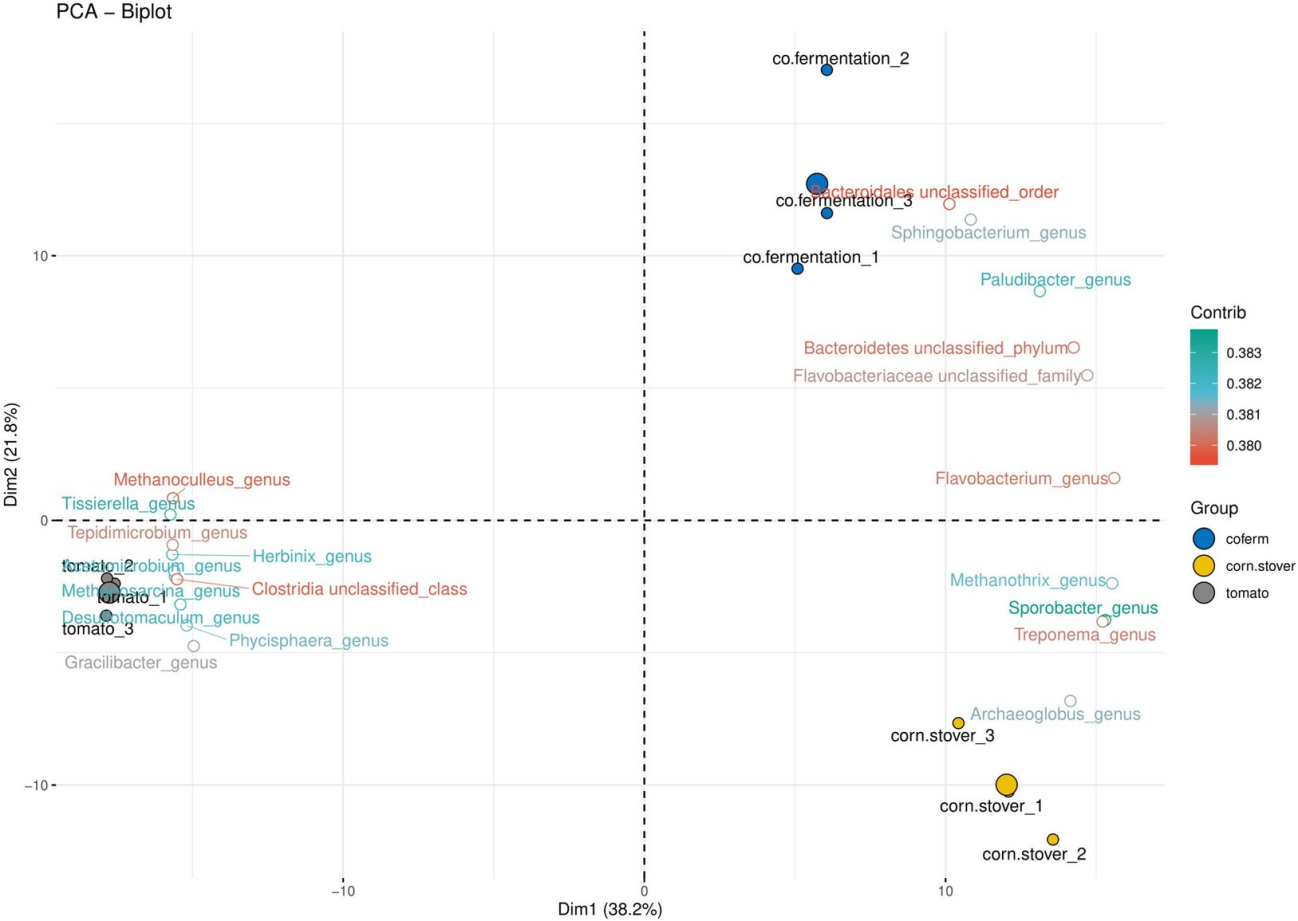
The Principal Component Analysis of CSTRs fed with CS (corn stover), TW (tomato waste), or CoS (co-substrate) at the genus level. The bigger, colored circles showed the average of technical parallels (1,2,3).

To further explore TW’s effect on the microbial compositions, the bacterial and archaeal communities were analyzed with Principal Component Analysis (PCA) (Fig 3). The two axes explained 60% of the total variance; the first axis explained 38.2%, and the second axis 21.8%. The CSTRs fed with TW, CS, and CoS formed diverse and discrete clusters. The parallel experiments were grouped together. As expected, the CoS samples were closer to the control (CS) than to the TW samples. The PCA results confirmed the influence of TW on the microbial community composition in AD.

First, the abundances of the bacterial and archaeal domains present in the various fermentations were compared to each other on the 40^th^ day. The microbial community of the inoculum was also investigated and presented as a reference. The bacterial domain predominated in all three continuous digestions, reaching 94.54% in abundance in CSTR_TW_, 94.97% in CSTR_CS_, and 95.72% in CSTR_CoS_. The archaeal domain followed with 5.46% in CSTR_TW_, 5.03% in CSTR_CS_, and 4.28% in CSTR_CoS_.

The most abundant bacterial phyla were Firmicutes, Bacteroidetes, Proteobacteria, Synergistetes, Spirochaetes, Actinobacteria, Chloroflexi, and Fibrobacteres; the read count of each phylum was higher than 0.7%. These phyla are found to be abundant in AD systems or biogas plants [48]. Firmicutes and Bacteroidetes were the most consistently dominant phyla in all samples. However, the relative read count of each sample was distinct. The Firmicutes accounted for 52.7%, 61.3%, and 48.3% of total reads in the CSTR_CS_, CSTR_TW_, and CSTR_CoS_ fermentations, respectively. The second most abundant bacterial phylum, the Bacteroidetes showed 26.7% (CSTR_CS_), 20.0% (CSTR_TW_) and 34.3% (CSTR_CoS_) appearance in the reactors.

The relative read counts of Synergistetes showed an increasing trend when fermenters were fed with TW or CoS; the abundances of this phylum increased by 5.14 and 1.5 times in CSTR_TW_ (8.9%) and CSTR_CoS_ (2.6%), relative to that of CSTR_CS_ (1.7%), respectively. Since several members of Synergistetes are known to ferment polypeptides and organic acids into acetate, hydrogen and carbon-dioxide, as well as to form syntrophic metabolism with hydrogenotrophic methanogens, an increase in their abundance in CSTR_TW_ was presumably due to the higher nitrogen content of TW (Table 1) [49, 50].

**Fig 4:**
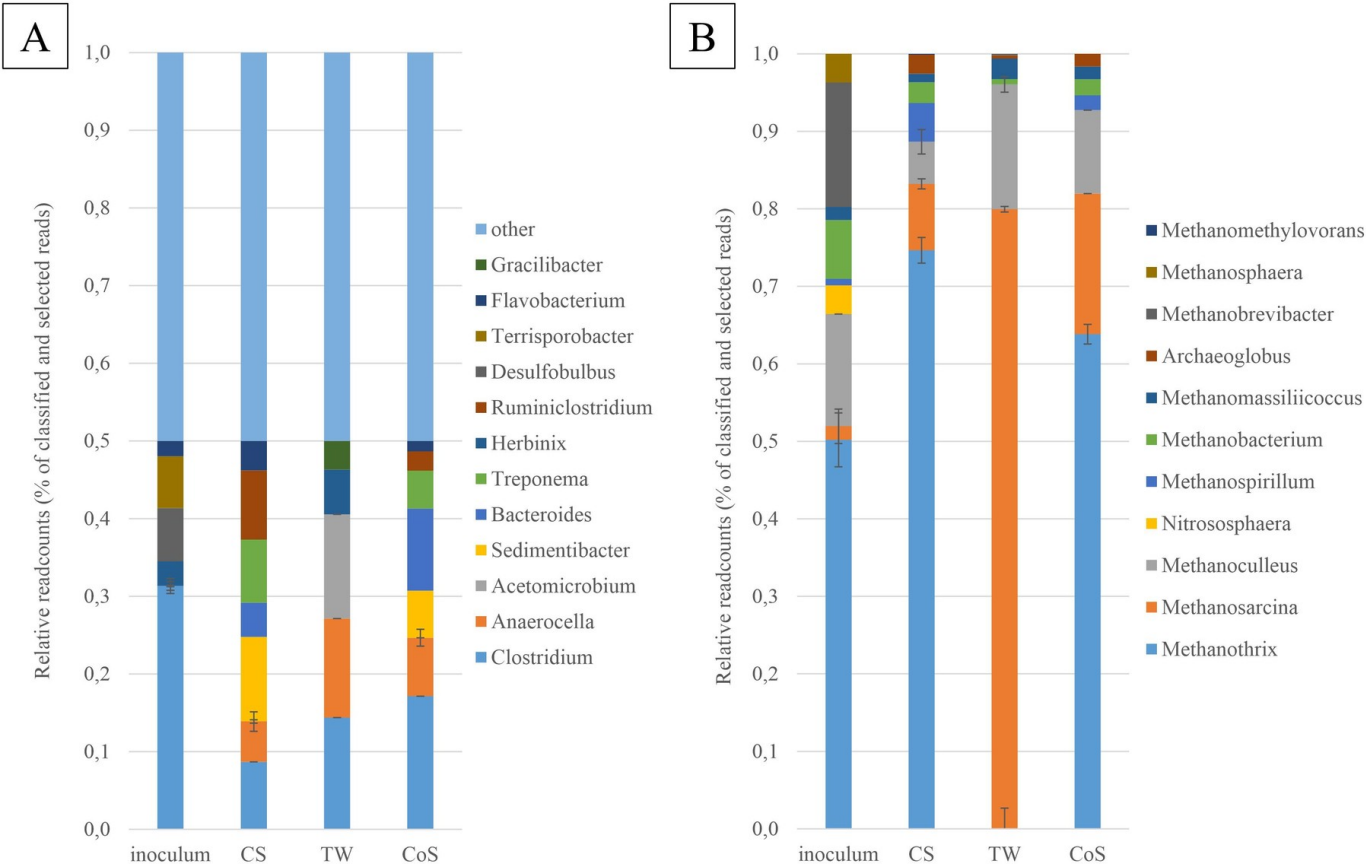
The relative read counts (% of classified and selected reads) of the bacterial (A) and archaeal domain (B) at the genus level. The top 11 phyla and genera belonging to the bacteria domain are presented. Bacterial genera with a relative read-counts lower than 3.0% were classified as others. All classified genera of the archaea domain are represented.

TW had the strongest effect on the microbial community composition in certain genera, such as *Clostridium* (of phylum Firmicutes), *Anaerocella* (Bacteroidetes), *Acetomicrobium* (Synergistetes), *Herbinix* (Firmicutes), and *Gracilibacter* (Firmicutes). The relative read counts of these genera were 1.88 (*Clostridium*), 2.80 (*Anaerocella*), 14.00 (*Acetomicrobium*), 6.00 (*Herbinix*), and 3.86 (*Gracilibacter*) times higher in the CSTR_TW_ than in the CSTR_CS_ fermentations (Fig 4A). The members of the *Clostridium* genus participate in the steps of hydrolysis, acidogenesis, and acetogenesis in AD [51, 52]. The members of the *Anaerocella* genus prefer carbohydrates, have a role in protein degradation and produce hydrogen and fatty acids [52]. The bacteria of the *Acetomicrobium* genus were formerly reported to ferment amino acids and saccharides to produce acetic acid and hydrogen [53]. Very little is known about the *Herbinix* and *Gracilibacter* bacteria; they were found to utilize saccharide monomers and produce VFAs and ethanol [16, 54].

Moreover, TW negatively affected the abundance of *Sedimentibacter* (Firmicutes), *Bacteroides* (Bacteroidetes), *Treponema* (Spirochaetes), and caused complete disappearance of *Ruminoclostridium* (Firmicutes) genus in the CSTR_TW_ fermentations (Fig 4A). Less pronounced, but similar tendencies could be observed in the composition of the microbial community in the CoS fermentations. The bacteria of the *Bacteroides* genus were previously described as plant cell wall degraders [55]. The bacteria of the genus *Ruminoclostridium* has a well-known role in cellulose degradation, and the *Treponema* species were found to degrade plant polysaccharides [56, 57]. The genus *Sedimentibacter* was also observed to be a core genus in household biogas digesters [58]. Taken together, the disappearance of cellulose- and hemicellulose-degrading bacteria from the CSTR_TW_ might have led to a decrease in biogas production.

#### Archaeal community

Only two archaeal phyla, Euryarchaeota and Thaumarchaeota, were detected. Euryarchaeota was the predominant phylum in all reactors. In contrast to the TW fermentations containing 0.7% of Thaumarchaeota, the occurrence of this phylum in CS and CoS fermentations was 3.2% and 4.2%, respectively. The Thaumarchaeota, a prevalent group in Archaea, is present in many ecosystems, including soil, biogas plants, and marine and fresh water [59, 60] and play important roles in both nitrogen- and carbon-cycles [61].

The distribution of archaeal sequences at the genus level shows that *Methanothrix*, *Methanosarcina*, and *Methanoculleus* were the predominant archaeal genera in both CSTR_CS_ and CSTR_CoS_, *Methanosarcina* and *Methanoculleus* were the most abundant taxa in the CSTR_TW_ fermentation, and *Methanothrix* was barely detectable (Fig 4B). At the same time, the relative read counts of *Methanosarcina* and *Methanoculleus* increased by 71.0% and 10.7%, respectively, as compared to that of CSTR_CS_ (Fig 4B). *Methanothrix* species are acetoclastic methanogens that utilize acetic acid to produce methane [62]; therefore, their disappearance is surprising and may be due to their inability to utilize hydrogen and carbon-dioxide as substrates for methanogenesis [63]. The genus *Methanosarcina* is able to utilize acetate, CO_2_, and H_2_ for methane production [48]. The genus *Methanoculleus* is hydrogenotrophic methanogen utilizing CO_2_ and H_2_ for methane production [64–66]. According to a previous study [67], methanogens undertake the hydrogenotrophic pathway under stressful conditions. Moreover, the high soluble organic content of the applied substrate could promote the hydrogen-utilizing methanogenes in the reactors [67]. From these data, it might be concluded, that—in TW fermentations—the biodiversity and the number of the dominant genera in the archaeal domain has decreased.

### Tomatine and tomatidine toxicity

**Fig 5:**
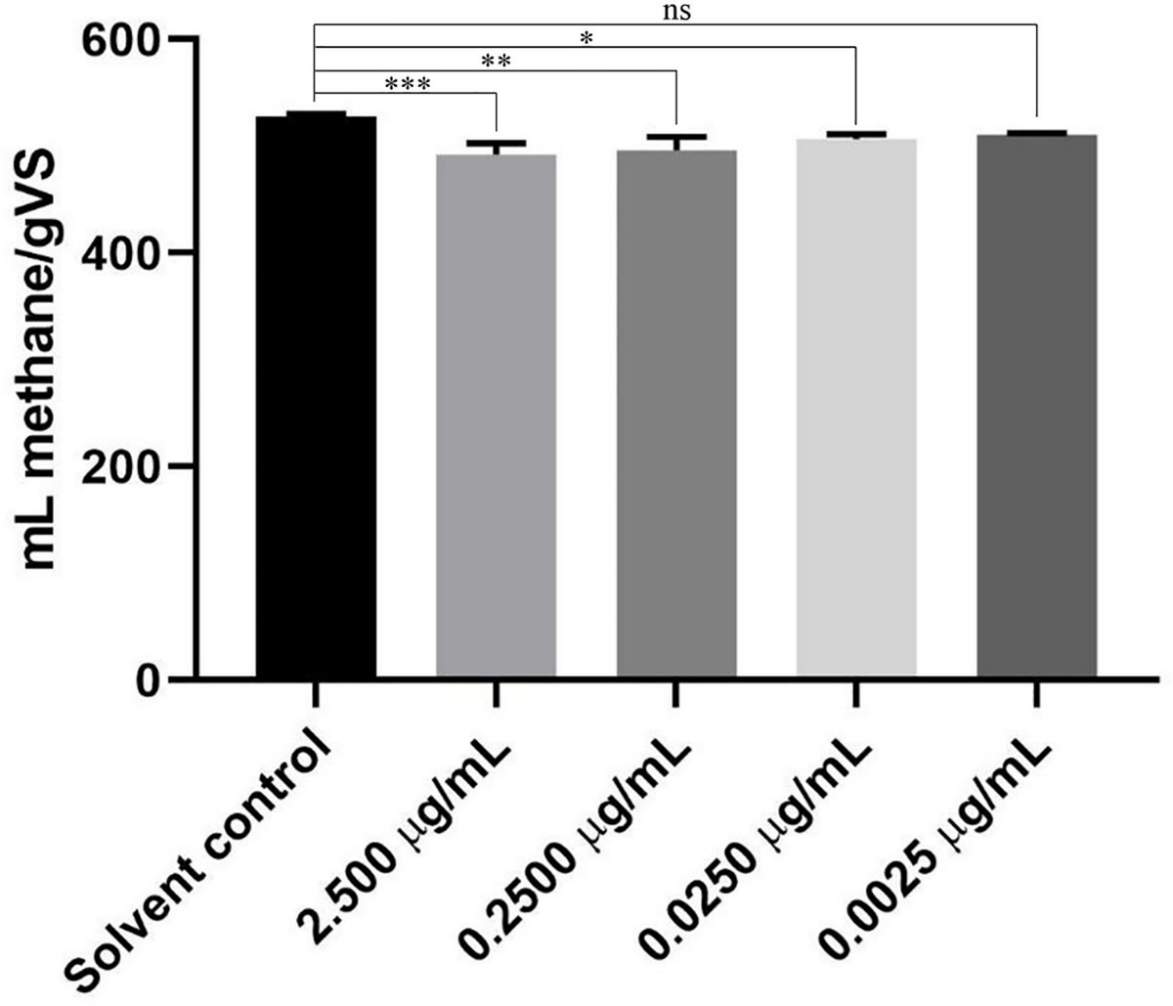
The effect of tomatine concentration on methane production in a batch experiment on day 16. The methane production of the reactors containg 2.5000, 0.2500, 0.0250, or 0.0025 μg/mL tomatine are shown to have significant differences, according to Dunnett’s multiple comparison test (n = 3, p ≤ 0.05).

TW has been found to contain bioactive compounds such as glycoalkaloids, i.e., tomatine and tomatidine, and phenols. Tomatine and tomatidine have been reported to have antimicrobial and antifungal effects under various conditions [9–11]; therefore, the effect of tomatine and tomatidine on batch fermentation was tested. Tomatidine, had no effect on the fermentation (The methane yields at 0.0000, 0.0025, 0.0250, 0.2500, 2.5000 μg/mL tomatidine concentrations were 523.7±2.8, 522.9±8.5, 524.0±6.2, 525.2±6.5, 523.7±7.0. In contrast, tomatine could inhibit the methane formation at 0.0250, 0.2500, 2.5000 μg/mL concentrations (Fig 5).

Tomatine’s negative effect was relatively stronger at the beginning of the fermentation; then, the effect gradually decreased until the end (S3 Fig). The decrease of the negative effect could be due to the microbial adaptation to tomatine (the time is likely too short) or tomatine’s decomposition during fermentation. Further analysis is necessary to address these possibilities.

### Phytotoxicity test

The evaluation of sewage sludge’s toxicity is important for assessing the applicability of the biogas plants’ effluents for agricultural purposes. Nowadays, phytotoxicity tests have attracted more attention and gained wider acceptance [41]. Seed germination and radicle length monitoring are the most common phytotoxicity tests. Hence, we determined germination indexes (GIs) of *Lactuca sativa L*. seeds and measured the radicle lengths (RLs) to assess the phytotoxicity of the various effluents from CS, TW, and CoS fermentations. Samples were taken from the CW-AD fermenters at the starting point, the midpoint, and the endpoint of the continuous fermentation experiments. GIs and RLs of lettuce were measured on the first, second, and third day. The effluent of CS, TW, and CoS fermentations, at 5%v/v effluent concentration, had no effect on the germination of lettuce seeds on the third day. On the other hand, the effluents applied at 5v/v% had minor negative effects on RL, while 3 v/v% effluents slightly increased the RL in all samples. Therefore, at 3 v/v%, none of the CW-AD effluents were phytotoxic on lettuce, and these effluents could be used in agriculture as a fertilizer.

## Conclusions

Tomato waste (TW), i.e., tomato stems and leaves, seems to be a promising substrate for biogas production since it has relatively low lignin and hemicellulose content and a lower C/N ratio than CS. Batch fermentation experiments confirmed that TW could be a promising substrate for biogas production both as mono-substrate or co-substrate with CS. However, in continuous AD systems, the involvement of TW decreased the biogas production. The fermentation parameters of the continuous AD experiments, such as pH, volatile fatty acid content, and NH_4_^+^ concentration, were optimal in all cases; thus, TW might have an inhibitory effect on the microbial community. The negative effect of tomatine on the biogas yield was confirmed in batch fermentation experiments, therefore TW should be applied as co-substrate in real applications.

The metagenomic analyses of the microbial composition in the fermentations revealed significant rearrangements, and reduced biodiversity in the microbial community in TW’s presence.

Agricultural usage of the fermentation effluent requires preliminary tests, including phytotoxicity assays. It has been demonstrated that the effluent of TW-containing fermentations had no toxic effect on *L*. *sativa var*. *capitata* germination.

The results demonstrated that tomato waste could be a good mono-substrate in batch fermentations or a co-substrate with corn stover in proper ratio in continuous anaerobic fermentations for biogas production. However, further optimization, including pretreatments or the application of thermophilic conditions, might further improve the process.

These results also point to the importance of running long-term continuous fermentations to test the suitability of a novel biomass substrate for industrial biogas production.

## Supporting information

S1 Fig A, Kinetics of the produced methane from corn stover (CS) and tomato waste (TW) in batch experiment. B, Cumulative methane production of CS and TW. There are not significant differences between the used substrates according to the Dunnett’s multiple comparison test (n = 3, p≤0.05).
(TIF)

S2 Fig α-diversity analysis for the fed-batch reactors fed with Corn Stover (CS), Tomato Waste (TW) and Co-Substrate (CoS). 3–3 technical parallel/reactor.
(TIF)

S3 Fig Effect of tomatine on the daily cumulative biomethane production. The tested concentrations were the following: 2.5000, 0.2500, 0.0250 and 0.0025 μg/mL.
(TIF)
