# Supplementary figures and images for: A comparative analysis of biogas production from tomato bio-waste in mesophilic batch and continuous anaerobic digestion systems
Source: PLoS One. 2021 Mar 17;16(3):e0248654. doi: 10.1371/journal.pone.0248654 (PMC7968646; doi:10.1371/journal.pone.0248654)

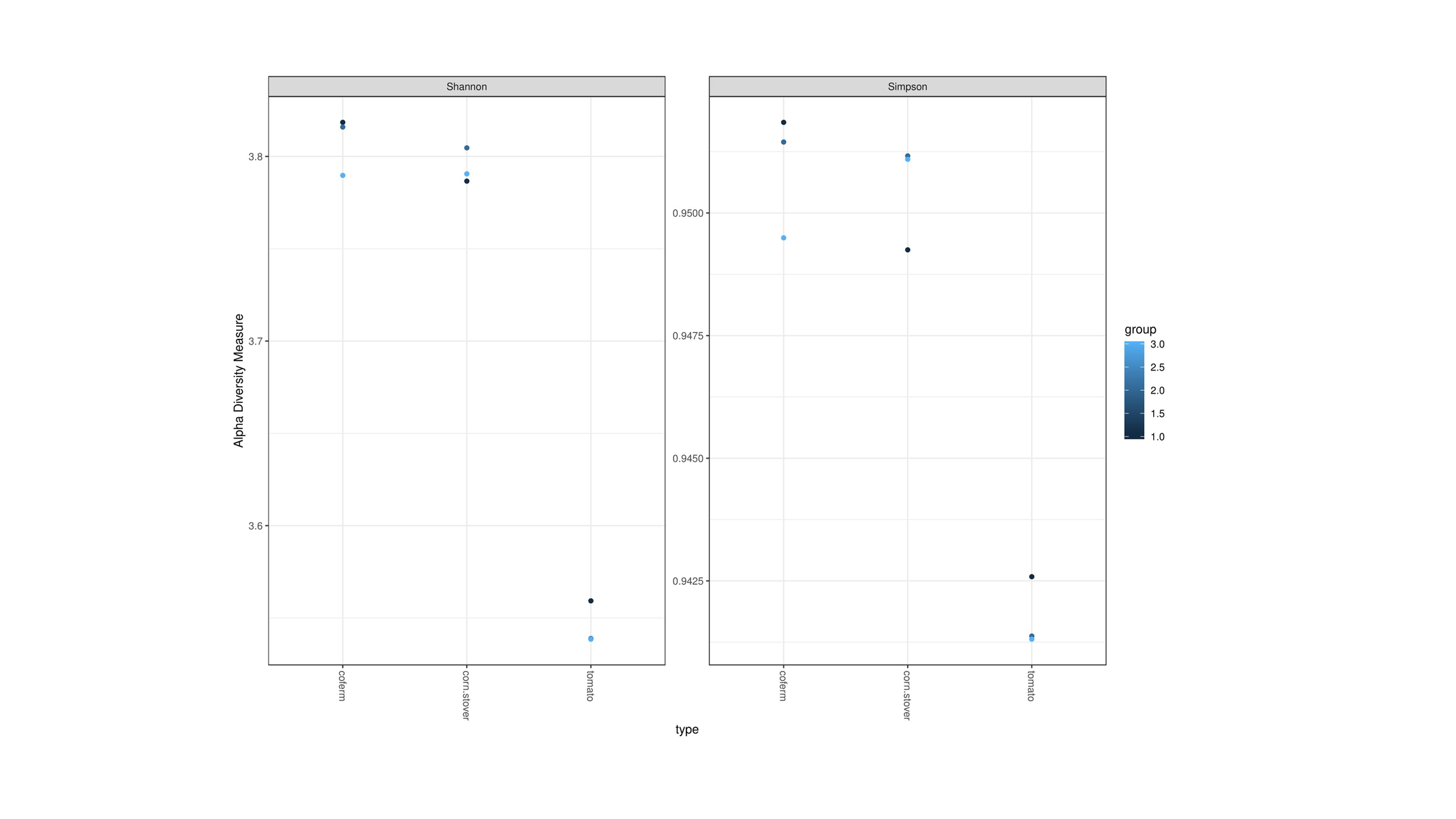

Supplement: S1 Fig — A, Kinetics of the produced methane from corn stover (CS) and tomato waste (TW) in batch experiment. B, Cumulative methane production of CS and TW. There are not significant differences between the used substrates according to the Dunnett’s multiple comparison test (n = 3, p≤0.05). (TIF) [file pone.0248654.s001.tif]

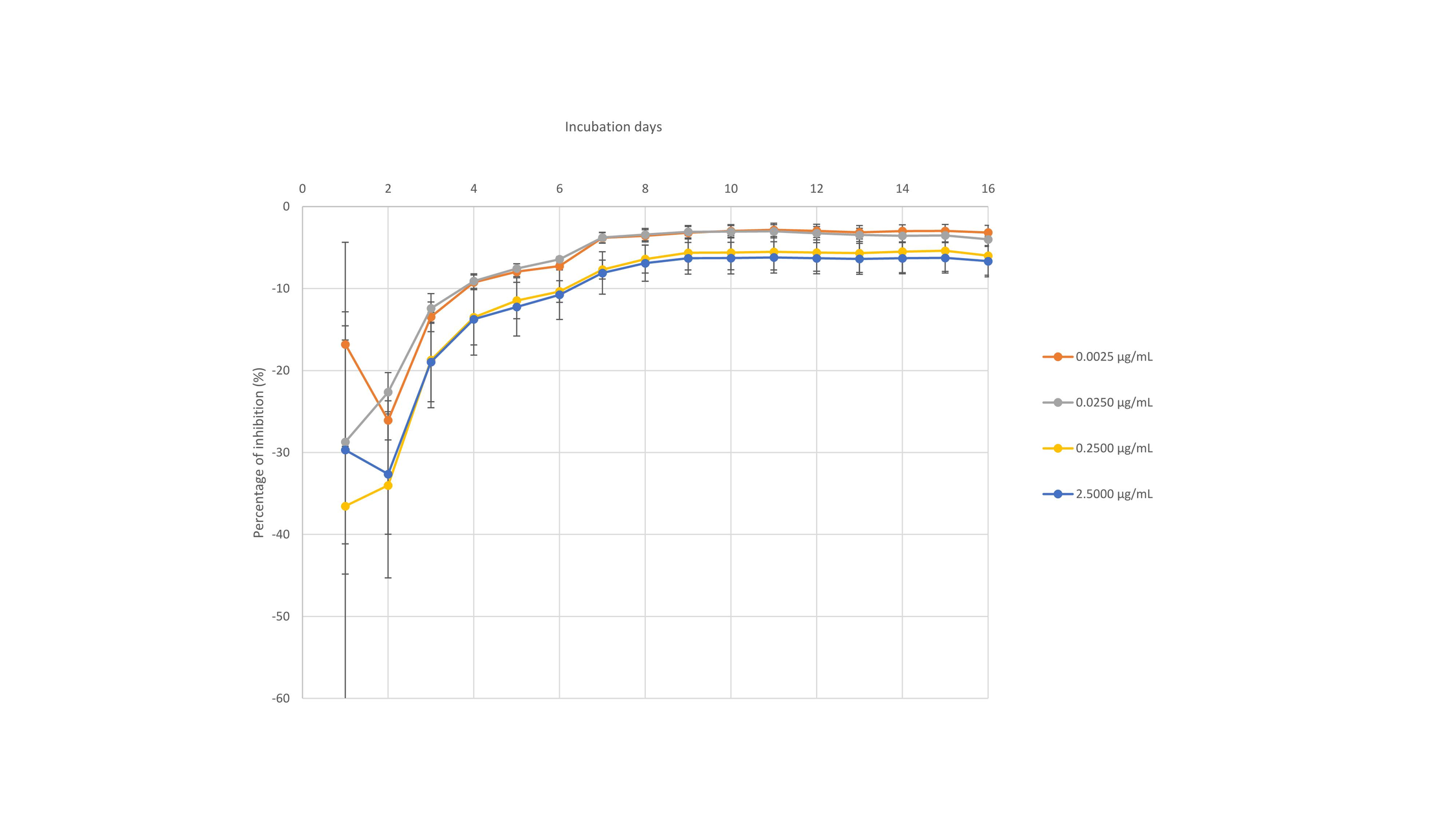

Supplement: S2 Fig — 3–3 technical parallel/reactor. (TIF) [file pone.0248654.s002.tif]
